# Postnatal Development of NPY and Somatostatin-28 Peptidergic Populations in the Human Angular Bundle

**DOI:** 10.3389/fnana.2018.00116

**Published:** 2019-01-08

**Authors:** Sandra Cebada-Sánchez, Pilar Marcos Rabal, Ana María Insausti, Ricardo Insausti

**Affiliations:** Human Neuroanatomy Laboratory, University of Castilla-La Mancha, Albacete, Spain

**Keywords:** human development, white matter, angular bundle, neuropeptide Y, somatostatin-28

## Abstract

The angular bundle is a white matter fiber fascicle, which runs longitudinally along the parahippocampal gyrus. It is best known for carrying fibers from the entorhinal cortex (EC) to the hippocampus through the perforant and alvear pathways, as well as for carrying hippocampal output to the neocortex, and distributing fibers to polysensory cortex. The angular bundle is already present prenatally at the beginning of the fetal period. Connections between the EC and the hippocampus are established by the 20th gestational week (gw). In the postnatal period, it shows increasing myelination. The angular bundle, as well as other white matter portions of gyral surfaces in the brain, presents interstitial neurons, a remnant of subplate neurons. Those interstitial neurons show neurochemical phenotypes both prenatally and postnatally, among which, neuropeptide Y (NPY) and Somatostatin-28 (SOM-28) peptidergic populations are noticeable, and accompany the fiber connections in the maturation of the hippocampal formation. We sought to investigate the topography of the postnatal distribution and relative density of neurons immunoreactive for NPY or SOM in the angular bundle along the rostrocaudal axis of the hippocampus. The study was carried out in 15 cases, ranging from 35 gws, up to 14 year old. All cases showed positive neurons showing a polygonal or spindle shaped morphology for both peptides, scattered throughout the angular bundle. The highest number of positive neurons appeared around birth and the ensuing weeks. Up to one and a half years, the density of both peptidergic populations decreased slightly. However, cases older than 2 years of age showed a substantial decrease in density of immunolabeled neurons, density that did not showed a minor decrease in density of positive neurons in cases older than 2 years. In addition, a topography from caudal to rostral levels of the angular bundle was detected at all ages. The functional significance of interstitial cells is unknown, but the existence of SOM and NPY peptidergic neurons, presumably inhibitory, in the white matter of the angular bundle, could contribute to the basic wiring of the hippocampal formation, through which autobiographical and spatial memories can begin to be stored in the infant brain.

## Introduction

The presence of neuropeptides in the white matter of the human brain has been known since 40 years. A number of studies demonstrate the existence of a variety of neurons containing several neuropeptides in the white matter. Among others, Neuropeptide Y (NPY), cholecystokinin (CCK), substance P (SP), Somatostatin-14 (SOM-14) or SOM-28 were detected (Chan-Palay et al., 1985, 1986; Chan-Palay, 1987; Del Fiacco et al., 1987; Lotstra and Vanderhaeghen, 1987a,b; Sakamoto et al., 1987; Van Reeth et al., 1987; Wahle and Meyer, 1987; Amaral et al., 1988; Lotstra et al., 1988, 1989; Cebada-Sánchez et al., 2014). Numerous peptide-immunoreactive fibers have also been described. While the origin and destination of the fibers forming those plexuses is at present unknown, the number does not seem to vary along the human postnatal development, and the relative neuron density apparently decreases with the infant brain growth (Lotstra et al., 1989).

Some of the reports alluded to above describe specifically neurons showing immunoreactivity for several peptides in the hippocampus and white matter underneath the hippocampus, as well as parahippocampal gyrus (which also includes the entorhinal cortex (EC), perirhinal and posterior parahippocampal cortices, see below for definition of the limits of the angular bundle white matter), including (Del Fiacco et al., 1987; Lotstra and Vanderhaeghen, 1987b; Lotstra et al., 1988, 1989; Quartu et al., 2001). The earliest detection of neuropeptide populations in the human central nervous system takes place at 5–7 weeks of gestation [enkephalin and SP, (Yew and Chan, 1999)], while immunoreactivity for NPY appears at 12 weeks in the spinal cord, and at 15 weeks in the hippocampus (Yew and Chan, 1999).

There is little information on the existence of interstitial neurons in the angular bundle in humans. The hippocampus itself appears around the 9th–10th gestational week (gw), at the beginning of the fetal period (Arnold and Trojanowski, 1996) albeit only in its dorsal division (González Arnay, 2015). The first indication of the presence of the EC in development takes place around the 10th gw (Kostović et al., 1993); it is not too far-fetched to suppose that some pioneer fibers start connecting both regions. Hippocampal subfields can be identified at 15th–19th gw (Arnold and Trojanowski, 1996). Hevner and Kinney (1996) showed that 10 gw later, that is, at the 20th gw, entorhino-hippocampal connections which travel in the angular bundle are established in human fetuses. The seminal work of Arnold and Trojanowski (1996) shows that all hippocampal subfields, including the dentate gyrus, are well formed by the 34th gw (8.5th month of gestation). There is little information on the angular bundle in humans, although it is known that it myelinates near birth, as has been demonstrated with both myelin basic protein (Arnold and Trojanowski, 1996) and myelin stain (Graterón Colmenares, 1997).

The angular bundle corresponds to the white matter tract placed in the ventromedial aspect of the medial temporal lobe, deep to the parahippocampal gyrus (*gyrus parahippocampalis*). While the angular bundle rests underneath the amygdaloid complex and the anterior part of the EC rostrally, caudally the angular bundle is limited dorsally by the Subiculum, Presubiculum and Parasubiculum on one hand, and the caudal part of the EC. The EC is followed caudally by the posterior parahippocampal cortex (mainly areas TH and TF of von Economo and Koskinas, 1927). Finally, at the level of the tail of the hippocampus and the parahippocampal isthmus (*isthmus parahippocampalis*), the angular bundle becomes narrower and courses behind the corpus callosum, to fuse finally with the posterior part of the cingulum bundle (*cingulum*).

The angular bundle is present in all mammal species, and since the studies of (Ramón y Cajal, 1904) and his disciple (Lorente de Nó, 1933, 1934), the angular bundle has been linked to the anatomical relationship between the EC, (*cortex entorhinalis*) and the hippocampus; Cajal himself linked the function of the EC to that of the hippocampus. Further studies with more modern tract tracing techniques not only confirmed this connection, but also elaborated a more detailed organization in the entorhino-hippocampal projection. In this way, the angular bundle carries a topographically organized projection from the EC to the *hippocampus* proper and in particular to the dentate gyrus (*Gyrus dentatus*). In all experimental species studied, in particular rodents (rat, mouse), the anterior part of the EC projects to the rostral hippocampus, while the caudal part of the EC projects to the posterior part of the hippocampus (Ruth et al., 1982, 1988; Insausti, 1993). Nonhuman primates follow this topographical arrangement of fibers; very likely humans follow the same pattern. Nonetheless, the collection of axons along the rostrocaudal extent of the angular bundle in humans is only very recently beginning to be unraveled (Kalus et al., 2006; Zeineh et al., 2017).

In nonhuman primates the angular bundle carries more fibers than just the entorhino-hippocampal projection (Lavenex et al., 2002). In fact, several bundles carry fibers from orbitofrontal and superior temporal gyrus distributing fibers to the EC (Insausti et al., 1987) and to perirhinal, inferotemporal and parahippocampal cortices as well (Insausti and Amaral, 2008). Fibers in the angular bundle do not only run in a rostrocaudal direction, but in a caudorostral direction as well; retrosplenial cortex afferents to the EC also run in the angular bundle, distributing fibers to parahippocampal and inferotemporal cortices (Kobayashi and Amaral, 2003; Lavenex et al., 2004). Moreover, EC projections to several cortical areas, mainly to the parahippocampal region (temporopolar, perirhinal and parahippocampal cortices) and other brain cortical areas such as the orbitofrontal, superior temporal gyrus, and the ventral part of the inferotemporal cortex, also travel for a variable stretch in the angular bundle (Legidos García, 2014). Other cortical areas of the parahippocampal region, mainly perirhinal and parahippocampal cortices distribute projections to a large variety of cortical areas (Lavenex et al., 2004).

However, to the best of our knowledge, no other connectional data on the angular bundle exist. More recent techniques as diffusion imaging tractography have explored the development of different fiber tracts along gestation (Song et al., 2015), but only indirect evidence from the cingulum bundle indicate that the ventral part of this tract reaches the parahippocampal gyrus.

Nowadays, peptidergic neurons of the white matter in the angular bundle and elsewhere are considered as interstitial neurons (Kostović and Rakic, 1980; Rakic, 2009; Kostović et al., 2014; Duque et al., 2016). NPY and SOM-28 interstitial neurons originate in the ventricular zone, and through radial migration, reach the subplate, along with layer VI neurons (Duque et al., 2016). Later on, subplate cells disperse by the action of thalamo-cortical, cortico-thalamic, basal forebrain and monoamine axons (Duque et al., 2016). Moreover, the subplate is wider at association cortices and at the gyral crown (the most external part of a gyrus). Although the subplate disappears postnatally, notwithstanding many cells remain, in particular in the white matter of the gyral crown, and in association cortices, as it is the case of prefrontal cortex (Kostović et al., 2011) and the parahippocampal gyrus (Insausti and Amaral, 2012). The dispersion of subplate neurons is a protracted phenomenon that can extend until the 2nd year of postnatal life (Kostović et al., 2014), and includes numerous neurons and fibers immunoreactive for NPY and SOM-28.

Interstitial white matter neurons immunoreactive for NPY are considered interneurons, both because of the shape (polymorphic near the crown of the giry and fusiform deep in the white matter) and size of peptide-immunoreactive neurons (Lotstra et al., 1989). Those interneurons are most abundant at birth and early postnatal years, and then decrease from 3 years or so; the number remains stable from 3 years to 4 years until adulthood, albeit the density decreases because of the brain growth (Lotstra et al., 1989).

We aim in this study at giving a more detailed account of white matter interneurons immunoreactive for two neuropeptides (NPY and SOM-28) in the angular bundle, as well as its distribution in the rostrocaudal extent of the angular bundle, a fiber tract that serves as pathway for important interconnections of memory related centers, such as the hippocampal formation and parahippocampal region (temporopolar, perirhinal and posterior parahippocampal cortices; see Insausti and Amaral, 2012 for concept of hippocampal formation), and Witter (2002) for that of parahippocampal region.

## Materials and Methods

The study is based in 15 human infant brains that were retrieved at different Neuropathology Departments in Albacete, Sevilla, Madrid and Pamplona (Spain), in the course of routine autopsies. Demographics, sex, age and cause of death are presented in Table 1. This study has been approved by the local Ethical Committee for Clinical Research of the University Hospital in Albacete (Spain), Session 10/06 and ISCIII-Red de Biobancos RD09/0076/00085, according to Spanish law and the Helsinki Declaration for medical research in humans. A neuropathological examination of all cases was performed by an expert neuropathologist; cases with no neurological diseases were only included in the study. The methodological procedure has been reported previously (Cebada-Sánchez et al., 2014), and only a brief summary will be presented here.

**Table 1:** Series of cases analyzed in this study.

Case	Sex	Gestational age at birth	Total age at death	Cause of death
1	NA	35 w	35 w	NA
2	Male	36 w	36 w	Diaphragmatic herniation
3	Male	37 w	37 w	ALA
4	Female	40 w	40 w	WME
5	Female	40 w	40 w	ALA
6	NA	40 w	40 w	TGV
7	Male	39 w	2 w	Bronchopheumonia (CHD)
8	Male	41 w + 6 d	4 w	CHD
9	Male	38 w + 3 d	6 w	CHD
10	Female	40 w	22 w	IC
11	Male	40 w	78 w	SDCO
12	Male	40 w	100 w	Sepsis
13	Male	40 w	261 w	Sepsis
14	Female	40 w	626 w	Hypovolemic Shock
15	Female	40 w	730 w	Lymphoma

*Sex, gestational age, postnatal age and cause of death are indicated. In two cases, the sex was not available (NA), but the age and cause of death are indicated. None of the cases presented any neurological disease. Abbreviations: gw, gestational weeks; d, days; h, hours; m, months; w, postnatal weeks; ALA, amniotic liquid aspiration; CHD, congenital heart disease; IC, intraventricular communication; SDCO, sudden death of cardiac origin; TGV, transposition of the great vessels; WME, without morphological expression*.

### Demographics

Table 1 shows the series analyzed in the present study. All cases were born at term (37–41 gw), except two cases that were somewhat younger (35 and 36 gw). None of the cases had neurological diseases or congenital malformations of nervous origin. There were eight males and five females (in two cases information about sex was not available). Postnatal ages ranged between less than 1 h and 14 years. The total age is expressed in postnatal weeks, even for the oldest cases, what offers a more uniform postnatal ages counted in weeks.

The brains were fixed in 10% formalin for a variable period of time, after which, and upon arrival to the Human Neuroanatomy Laboratory were transferred to a 4% paraformaldehyde in phosphate buffered saline (PBS, pH 7.4) for at least 1 month (Insausti et al., 2010). After blocking the brains (Cebada-Sánchez et al., 2014), the different blocks containing the entirety of the hippocampus were immersed into a cryoprotectant solution in increasing graded sucrose solutions (15%–30% in PBS) until they sank. Blocks were serially cut in the coronal plane on a sliding microtome coupled to a freezing unit (Micron, Heidelberg, Germany). The section thickness was 50 μm. Every 250 μm (one-in-five), sections were Nissl-stained with 0.25% thionin. Adjacent sections were used for immunohistochemical detection of NPY or SOM-28 at three different levels of the angular bundle (taking the EC and hippocampus as reference): (1) level of the uncus; (2) mid-level; coincident with the beginning of the hippocampal body; and (3) caudal at level of the hippocampal tail. Sections included the complete parahippocampal and fusiform gyri, so that the whole extent of the angular bundle in the parahippocampal gyrus was included in the study, and as far as the isthmus of the parahippocampal gyrus. Part of this material has been previously used in Insausti et al. (2010) and Cebada-Sánchez et al. (2014).

### Immunohistochemical Procedure

The general procedure of immunohistochemical staining has been reported previously (Cebada-Sánchez et al., 2014). In brief, all series of NPY and SOM-28 immunostaining were carried out simultaneously for each group of rostrocaudal levels of the angular bundle and ages. Sections were treated for elimination of endogenous peroxidase activity (Guntern et al., 1989). After several washes in PBS a preincubation was performed in a PBS solution containing 1% normal horse serum, 1% bovine serum albumin (BSA), and 0.3% Triton X-100. Anti-NPY primary antibody, raised in rabbits, was kindly donated by Professor G. Tramu (Université de Bordeaux I, France), and was used at a dilution of 1:5,000. Goat anti-SOM-28 antibody (Santa Cruz Biotechnology, CA, USA) was used at a dilution of 1:1,000. Sections were incubated separately for each primary antibody at 4°C overnight. Afterwards, sections were incubated with the corresponding biotinylated secondary antibodies (dilution 1:2,000; Jackson Immunoresearch Europe, Suffolk, UK) for 90 min, then with peroxidase-coupled streptavidin (same dilution and time than secondary antibodies). Thereafter, immunohistochemical reaction was developed by means of 3-3′-diaminobenzidine method. Sections were weakly counterstained with thionin, dehydrated, and coverslipped with DPX. The specificity of the antibodies has been dealt with in a previous report (Cebada-Sánchez et al., 2014).

Immunoreactive cells were identified under microscopic examination and their location charted at the three rostrocaudal levels alluded to above (rostral, mid, caudal) by means of a computerized stage system in all cases (AccuStage, Minnesota Datametrics MD-3 Digitizer, software MDPLOT v4). This program also allowed the calculation of the density of immunolabeled neurons per square millimeter, what was used in the graphical representation of densities for both peptides across ages. In delineating the precise extent of the white matter neurons areas in which neuron density was calculated, we used a similar protocol as in Mortazavi et al. (2017).

Images were obtained with a Nikon Eclipse 80-I microscope coupled to an image capture system (Application suite V.4). The images were adjusted only for contrast and brightness with Adobe Photoshop CC (2017), without any other modification. Schematic representation of the charted cells was prepared with Canvas X (Deneba Systems, Florida, FL, USA).

## Results

### Limits of the Angular Bundle Determination in the Medial Temporal Lobe

The angular bundle corresponds to the white matter in the parahippocampal gyrus. The boundaries change along the rostrocaudal axis: rostrally, the upper limit is the amygdaloid complex; layer VI of the EC is the medial boundary, and the white matter lining the cortex of the medial bank of the collateral sulcus is the lateral boundary.

At the point where the Subiculum starts, the upper boundary is formed by CA1, Subiculum and Presubiculum in a lateral to medial sequence; the medial, ventral and lateral boundaries are the EC (plus Parasubiculum) and medial bank of the collateral sulcus respectively. The medial limit formed by layer VI of the rostral portion of the EC does not change for most of the EC extent, although the border white matter/gray matter is more distinct caudal to the beginning of the hippocampal fissure (Insausti et al., 1995). Therefore, the angular bundle, anterior to the caudal tip of the uncus (*Gyrus intralimbicus*), is covered by the gray matter of the entorhinal and perirhinal^1^ cortices (the latter only at the medial bank of the collateral sulcus, which includes the transentorhinal cortex, perirhinal cortex or Brodmann’s area 35, and less often, ectorhinal cortex or Brodmann’s area 36 (Brodmann, 1909). Caudal to the *Gyrus intralimbicus*, the angular bundle is surrounded dorsally, by CA1, Subiculum and Presubiculum; medially; laterally by the Parasubiculum and areas TH and TF of von Economo and Koskinas (1927), which together make up the posterior parahippocampal cortex. The posterior end of the angular bundle is indistinct, as the angular bundle is continuous with the white matter of the cingulum bundle, which arches around the corpus callosum to meet the white matter of the isthmus of the parahippocampal gyrus. In fact, some authors (Brody et al., 1987; Mai et al., 2016), instead of naming the white matter of the parahippocampal gyrus as angular bundle, they rather use the term cingulum bundle. Regardless of the nomenclature, all reference is to the white matter of the parahippocampal gyrus.

The lateral part of the angular bundle is continuous with the remainder of the white matter of the temporal lobe, which corresponds to the confluence of the inferior, middle and superior temporal gyri white matter. Therefore, the lateral limit of the angular bundle is arbitrary, although in our study we placed the lateral part of the angular bundle at the collateral eminence (*eminentia collateralis*), a prominence that the collateral sulcus makes at the ventrolateral part of the temporal horn of the lateral ventricle.

### Shape and Overall Density of NPY-Immunoreactive Cell Bodies in the Angular Bundle

Large amounts of NPY-immunoreactive cell bodies and fibers were found in the angular bundle near the time of birth. NPY-positive cell bodies were scattered throughout the extent of the angular bundle without any regular distribution.

**Figure 1:**
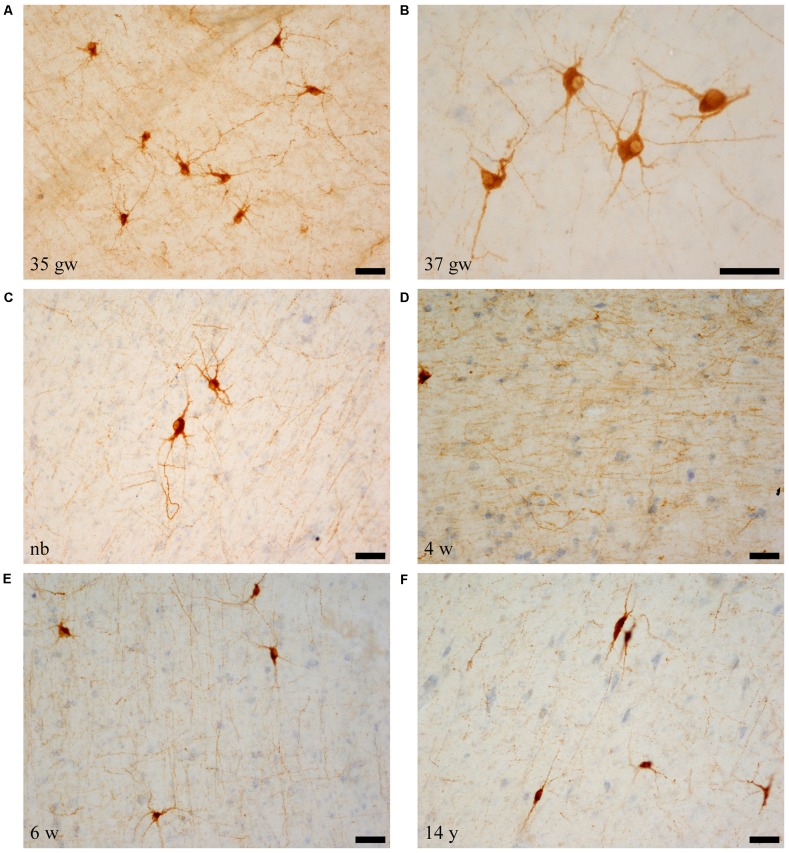
Microphotographs of neuropeptide Y (NPY)-immunoreactive neurons and fibers at different ages. Panels **(A–C)** are the youngest cases (35–40 weeks), which show maximal amount of neuron and fiber immunoreaction. Most NPY-positive neurons are polygonal in shape. Panel **(D)** shows NPY-immunoreactive fibers, and panel **(F)** shows spindle shaped neurons at 14 years (730 weeks). In relation to the topography of the hippocampus, **(A,D,F)** are from rostral levels, **(C)** is midlevel, and **(B,E)** are at caudal levels. Scale bar is 50 μm.

Immunolabeled cells had the appearance of neurons, and none of the NPY labeled cells seemed to present glial morphology in our preparations (i.e., Figures 1A–C). Neurons showed two main types of shapes: the first was polygonal or triangular, displaying thick dendrites with short and stout ramifications (Figures 1A–C); the other shape was elongated, less abundant with a smaller soma size, and morphology of a bipolar neuron (Figure 1F). The appearance of NPY-positive cells did not change along our series of ages (Figure 1). However, a trend was observed for the complexity of the dendritic tree revealed in the immunoreaction: the younger the case, the more extensive the visible dendritic tree (Figure 1, compare panels A,B and panel F). The size of the immunoreactive cells also seemed to decrease with age (Figure 1, compare panels A and C with panel F, all at the same magnification). We could not appreciate axons that could be followed for some distance.

The immunostained preparations also showed a great amount of stained fibers which formed a dense meshwork throughout the angular bundle (Figure 1D).

**Figure 2:**
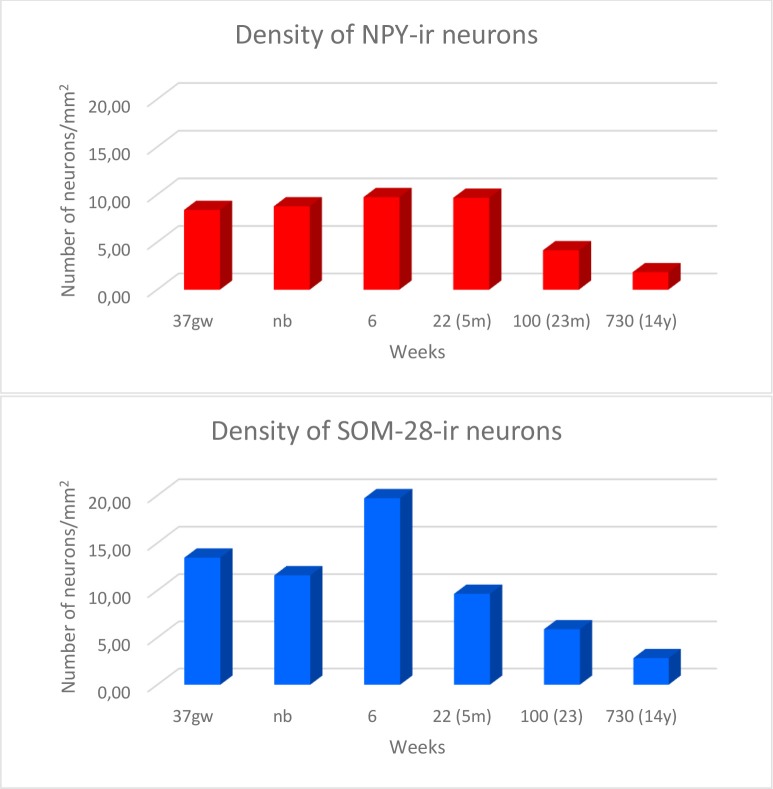
Density of NPY- (in red) and somatostatin-28 (SOM-28)- (in blue) immunoreactive neurons expressed as units per square millimeter across different ages as determined at midlevel of the angular bundle.

**Figure 3:**
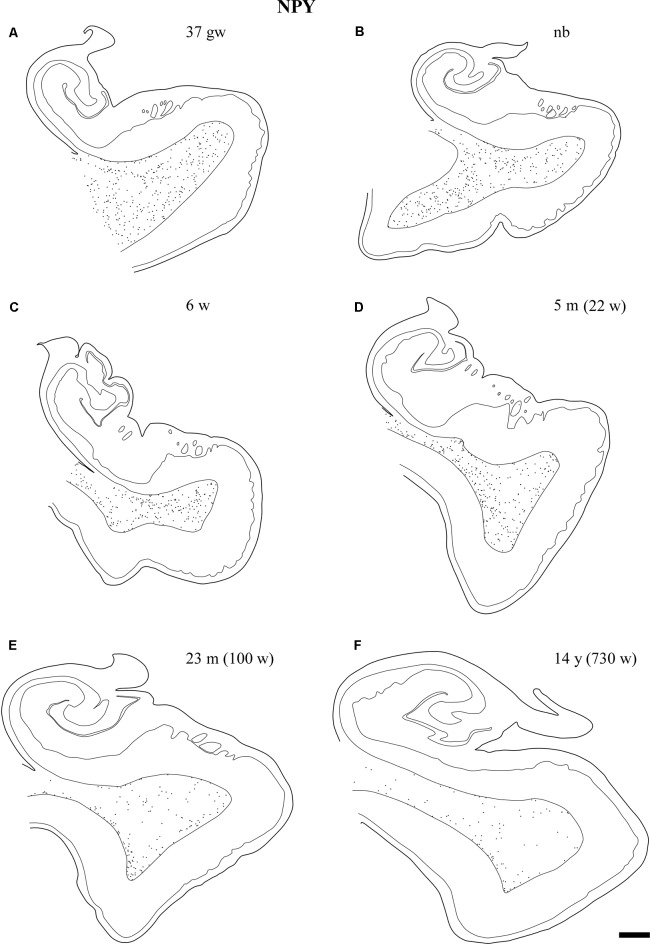
Line-drawings **(A–F)** of the angular bundle white matter at midlevel of the hippocampus across ages in which NPY-positive neurons have been plotted. Individual neurons are represented by single dots. Other labeled neurons outside the angular bundle have not been represented. The age is indicated on each drawing. Note the decrease in the density of NPY-positive neurons with age. Scale bar is 2 mm.

Overall, the number of NPY-positive cells was higher around the time of birth. The number of cells seemed to be rather invariant in the youngest cases, although apparently, the neuron number showed a slight decrease around 2 years, in our series at 23 months (Figure 2). The density of positive cells in our material did not vary much despite the growth of the brain during the first 2 years (Insausti et al., 2010). We found that a decrease in the number of NPY-positive neurons in the angular bundle begun to decrease from 23 months on (100 postnatal weeks, almost two postnatal years, Figure 2). This did not seem to occur in ages younger than 23 months. Although we could study only three cases older than 23 months (5, 12 and 14 years), the trend towards a progressive reduction in the number of NPY positive cells was consistent. Figure 3 presents charts of NPY-immunoreactive cells in the angular bundle at mid-level, from 37 gw to 14 years (730 weeks). In this illustration panels E (case of 23 months) and F (case of 14 years old) show a clear decrease in NPY-positive cells compared to panels A-D which correspond to younger ages.

The location of NPY-positive neurons in two specific places deserves special mention because of the particular orientation of cells and fibers. The first one is at the crown of the angular bundle, abutting the Parasubiculum both superior and medially, and inferiorly towards the medial shoulder of the collateral sulcus (see boundaries of the angular bundle above). Here, NPY-positive cells presented bipolar or fusiform neurons amidst a loose meshwork of NPY-positive fibers, which took a radial orientation pattern, fanning out from the lateral limit of the angular bundle, among which, bipolar or fusiform NPY-positive neurons followed also the same orientation. This organization of cells and fibers was more evident at the time of birth, but it also continued in subsequent ages up to our oldest age (14 years).

The second one lies at the vicinity of the temporal horn of the lateral ventricle. This zone occupies the raphe in continuation of the fused walls of the closed ventricular cavity, which extends medially under distal CA1 and proximal part of the Subiculum. This region contained numerous NPY-positive neurons, in particular at young ages (Figure 3). The NPY-immunoreactive neurons were mainly bipolar, whose long axis was parallel to the direction of this line of fusion of the ventricular walls. Of note, the presence of this group of neurons decreased at about 5 months of age and could not be observed at the oldest ages (12 and 14 years).

### Shape and Overall Density of SOM-28-Immunoreactive Cell Bodies in the Angular Bundle

**Figure 4:**
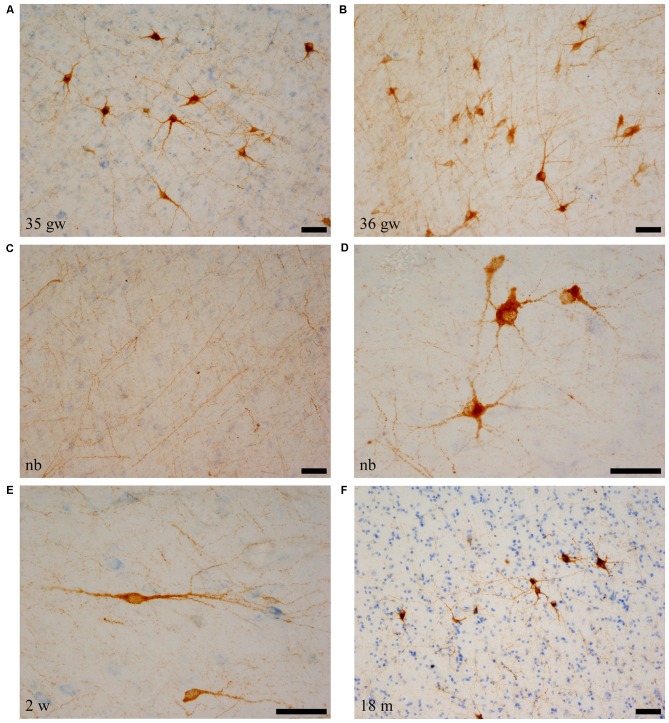
SOM-28-positive neurons and fibers in the angular bundle white matter. Note the polygonal (panel **D**, newborn, case 5), spindle-shaped neurons (panel **E**, case 7, 2 weeks), or mixed morphology population **(A,B,F)**. Fibers containing SOM-28 are presented in panel **C**. Note that the youngest ages show the most detailed morphology. Microphotographs **(A,E)** correspond to rostral levels of the hippocampus; Pictures **(B–D,F)** are at midlevel of the hippocampus. Scale bar is 50 μm.

**Figure 5:**
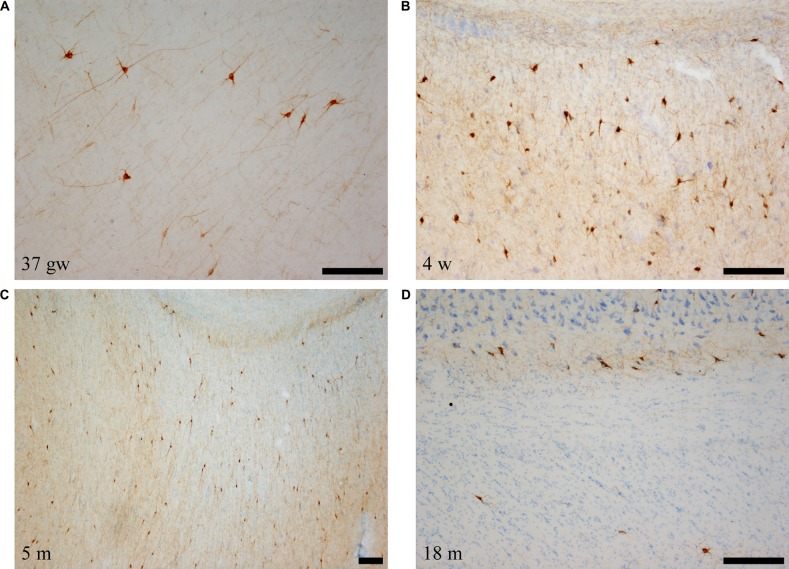
Low power photomicrographs of the distribution of SOM-28-immunoreactive neurons in the angular bundle at increasing ages. Panel **(A)** is a 37 gestational week (gw) case showing multipolar, neurons, dendrites as well as immunostained fibers deep in the angular bundle. Panel **(B)** shows the concentration of stained neurons at the border with the entorhinal cortex (EC; to the right in the panel). Panel **(C)** shows that the density of labeled neurons is still high at 5 months, while panel **(D)** shows that, by 18 months, the apparent density is clearly lower. Topographically, panels **(B,C)** are at the rostral portion of the angular bundle; panels **(A,D)** correspond to caudal levels of the hippocampus. Scale bar is 200 μm.

**Figure 6:**
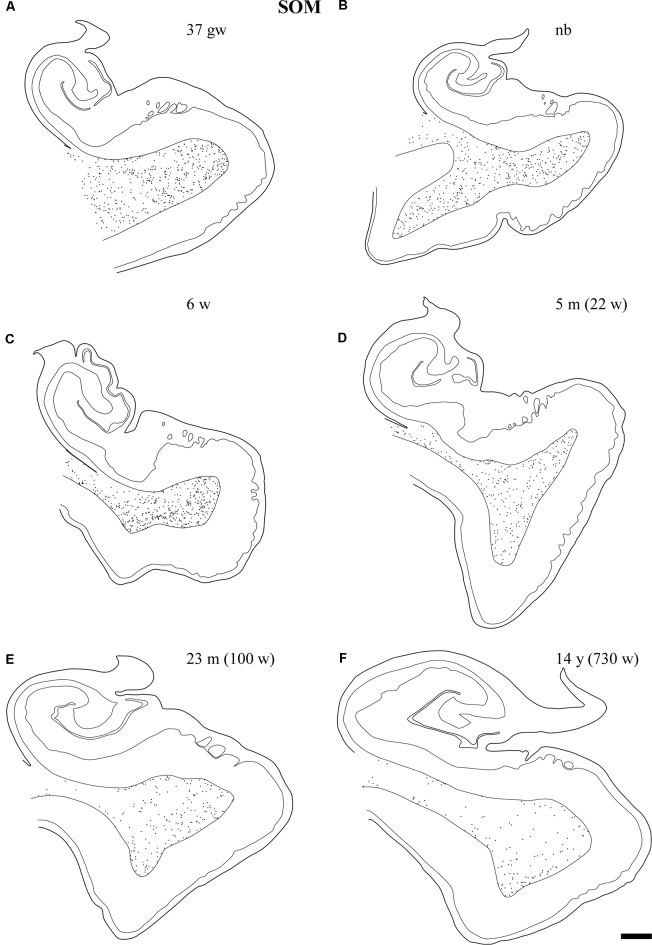
Line drawings with representation of a series of sections of the angular bundle at midlevel in which SOM-28 neurons have been plotted. Only white matter neurons were charted. Panels **(A,B)** shows high density of SOM-28-immunolabeled neurons, which decrease by 23 months **(E)** and continues into 14 years **(F)**. Other conventions as in Figure 3. Scale bar is 2 mm.

As it happened in the case of NPY immunoreactivity, numerous SOM-28-positive neurons were found in the angular bundle at all ages examined. Also, and similar to the case of NPY immunoreactivity, the labeled cells had the appearance of neurons rather than glia (astrocytes) in our preparations (Figures 4A,B,D–F). The shape of neurons containing SOM-28 was in most cases multipolar; bipolar neuron morphology appeared although to a lesser extent (Figures 4D,E, 5). A rather dense meshwork of positive fibers also populated the white matter of the angular bundle among positive neurons (Figure 4C). Immunoreactive neurons were particularly numerous around six postnatal weeks (Figure 6). The shape of the SOM-28-positive neurons did not change with age, although neurons had shorter, more stout dendrites with increasing age (Figure 4F).

The number of SOM-28-immunoreactive positive neurons was higher relative to NPY-positive neurons at similar ages, as it was observed in the hilar region of the dentate gyrus (Cebada-Sánchez et al., 2014). The relative density of labeled neurons was clearly denser in the cases at young ages, that is, perinatally (Figure 5), while cases at more advanced ages showed a decrease in the relative density of SOM-28-positive neurons (Figures 6E,F).

The population of neurons containing SOM-28 showed at some points some peculiarities in the location and arrangement of immunoreactive neurons and fibers, similar to that observed in the NPY series. The first point was at the gyral crown of the angular bundle. There, SOM-28-positive neurons and fibers were oriented radially in the white matter of the angular bundle. They were directed towards the crown of the gyral surface of the parahippocampal gyrus, even more distinctively than NPY-immunoreactive preparations.

The second point was, near the lateral ventricle, as it was the case in the NPY series. Specifically, a population of SOM-28 positive neurons was observed near the fused walls of the lateral ventricle, underneath the alveus of distal CA1 and deep layers of the proximal Subiculum (Figure 6). In contrast to the NPY population observed at the same place, both bipolar neurons and multipolar neurons populated this region.

Our series showed abundant NPY-immunoreactive neurons at all ages extended and randomly distributed in the angular bundle white matter (Figure 3). The highest density corresponded to the late weeks of pregnancy (Figure 3A) as well as at birth (Figure 3B), although they did not show any specific pattern of distribution. At 6 and 22 weeks of postnatal age little change occurred (Figures 3C,D). However, by 23 months (100 postnatal weeks, Figure 3E) and 14 years (730 postnatal weeks, Figure 3F), the overall density decreased substantially; in particular by 14 years (730 postnatal weeks).

NPY-positive neurons were relatively more abundant close to the border with the gray matter, in a loose boundary with layer VI of the cortical fields that surrounded the angular bundle (see above boundaries of the angular bundle). In contrast, fewer NPY-immunoreactive neurons were observed in the depths of the angular bundle.

The overall density of SOM-28-positive neurons was higher relative to NPY positive neurons (compare Figures 3, 6). This feature was present for all ages and at all levels. However, the overall distribution in both cases was very similar. Scattered positive neurons without any specific distribution were visible in all parts of the angular bundle. High numbers of SOM-28-positive neurons were present at 37 gw (Figure 6A), time of birth (Figure 6B), six postnatal weeks (Figure 6C) and 5 months (22 postnatal weeks, Figures 2, 6D). A noticeable decrease in the density of labeled neurons was observed at 23 months (100 postnatal weeks, Figures 2, 6E), but at 14 years (730 postnatal weeks), the reduction of SOM-28-positive neurons is even more clear (Figures 2, 6F). Topographically, the positive neurons showed higher density at the periphery of the angular bundle white matter (Figure 6F). Notice that all line drawings of sections with plots of SOM-28-positive neurons are represented at the same magnification.

### Longitudinal Angular Bundle Distribution of NPY- and SOM-28-Immunoreactive Neurons

**Figure 7:**
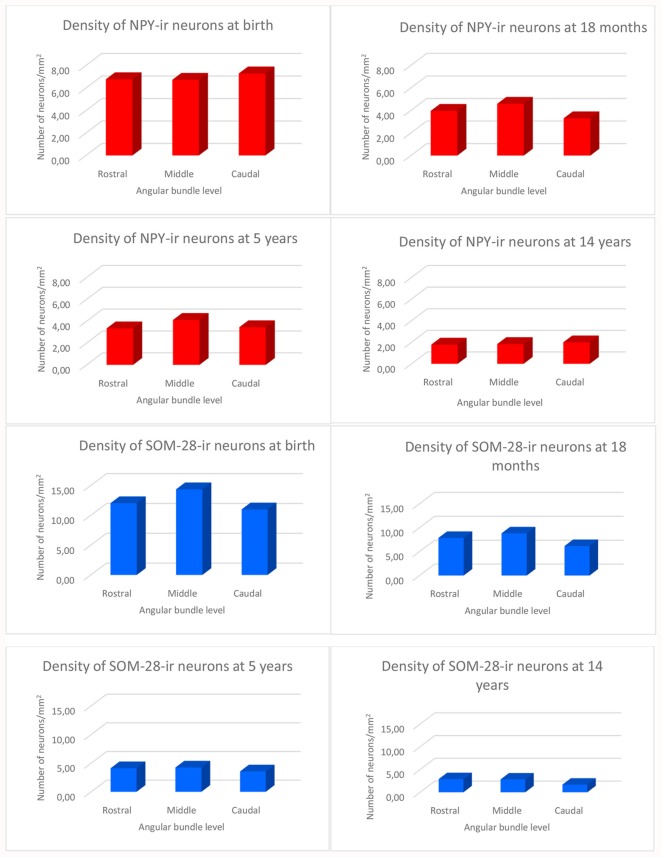
Histograms showing the density of NPY and SOM-immunoreactive neurons per square millimeter distributed at three levels of the angular bundle: rostral, mid and caudal. Note the different scale for the number of labeled neurons used in NPY and SOM neuropeptides, respectively.

**Figure 8:**
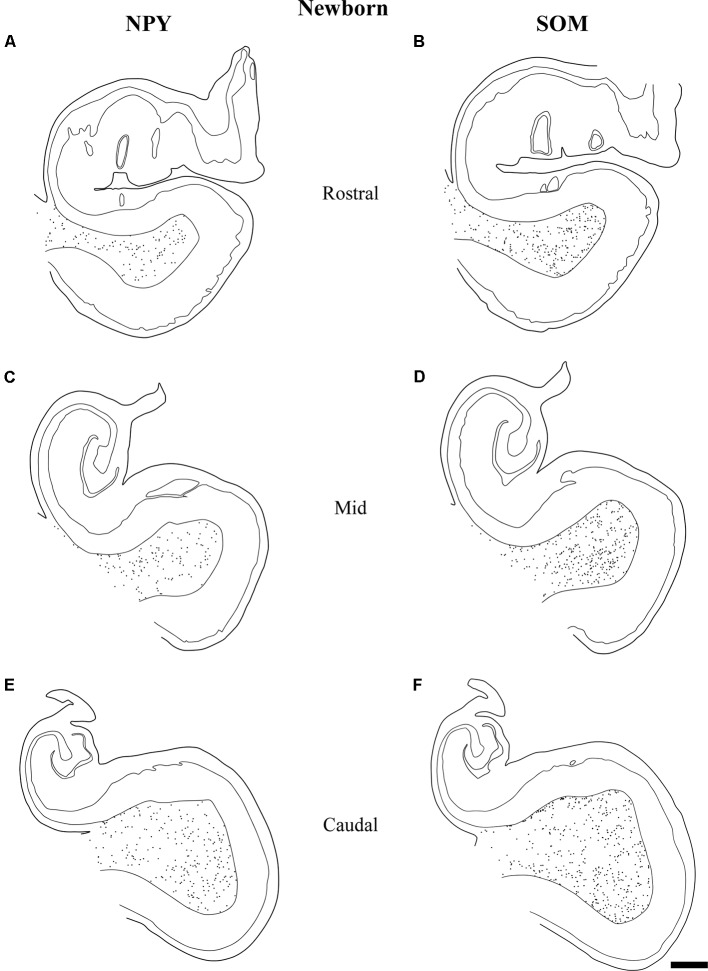
The variation in density of both NPY and SOM-28 neurons at birth is depicted at three rostrocaudal levels of the angular bundle studied. Note the overall high, homogeneous density of labeled neurons for both neuropeptides in the angular bundle at the three levels. Other conventions as in Figure 3. Scale bar is 2 mm.

**Figure 9:**
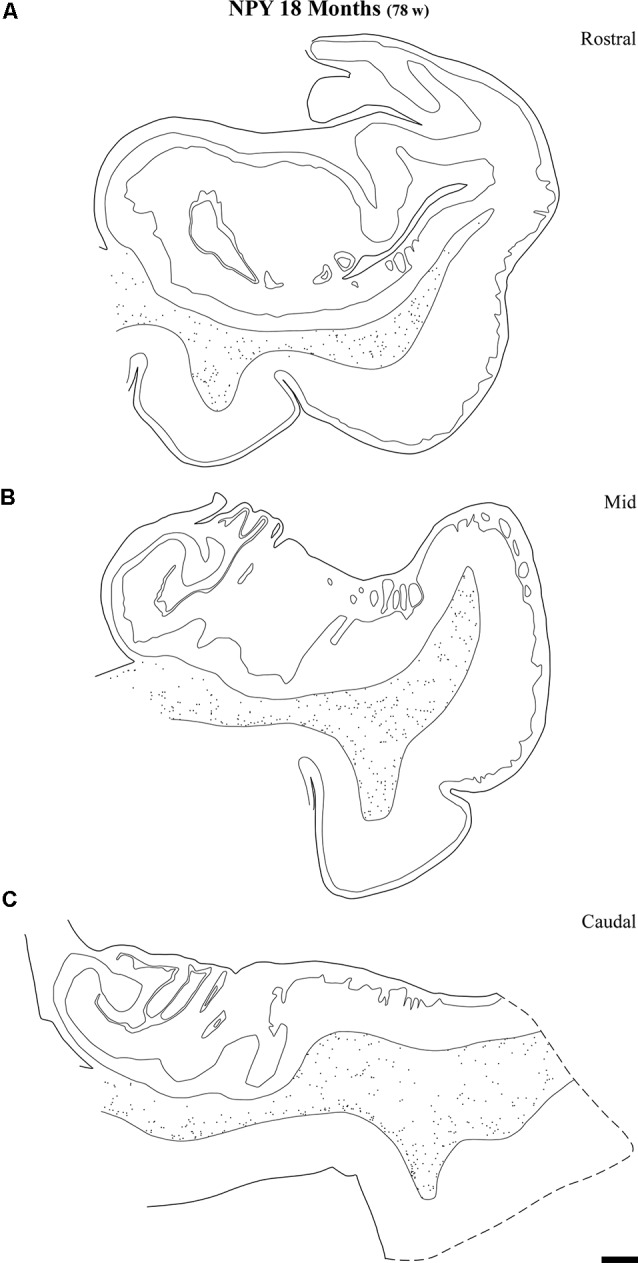
Line drawings of charted NPY-positive neurons at three different rostrocaudal levels of the angular bundle at one and a half years (18 months). While the rostral **(A)** and mid-level **(B)** of the series contains a substantial number of labeled neurons, the caudal section **(C)** shows fewer labeled neurons. Other conventions as in Figure 3. Scale bar is 2 mm.

**Figure 10:**
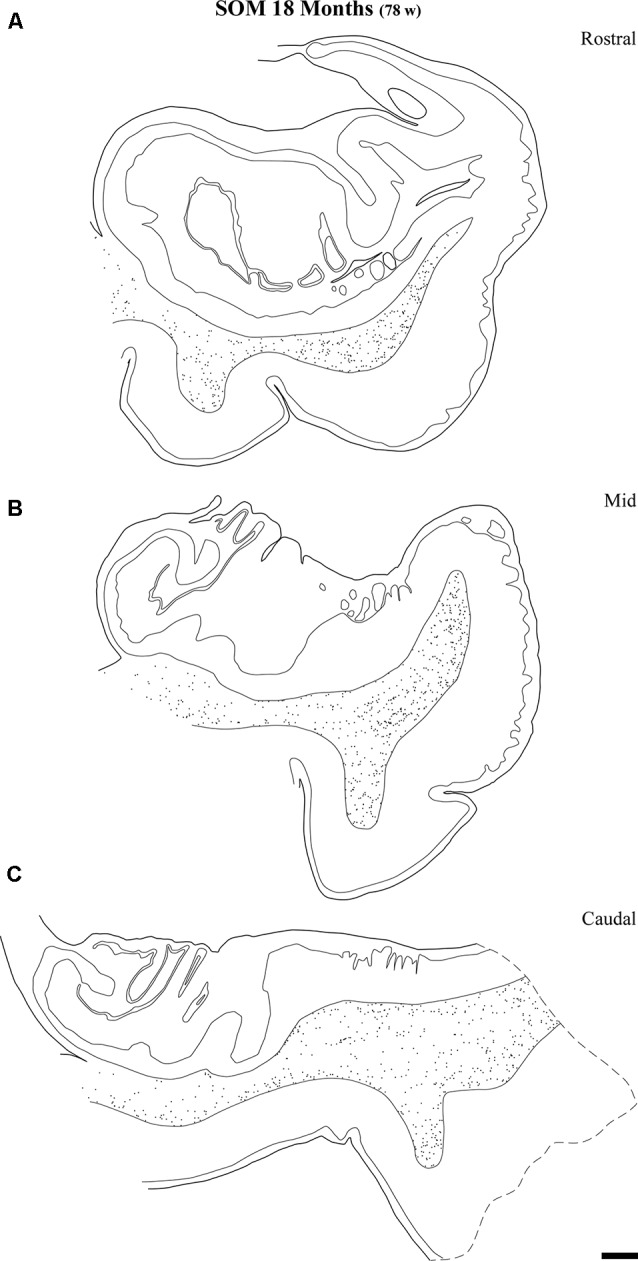
Distribution of SOM-28-positive neurons at three rostrocaudal levels of the angular bundle at 18 months. Note the relative higher density rostral at midlevel. Conventions as in Figure 3. Scale bar is 2 mm.

**Figure 11:**
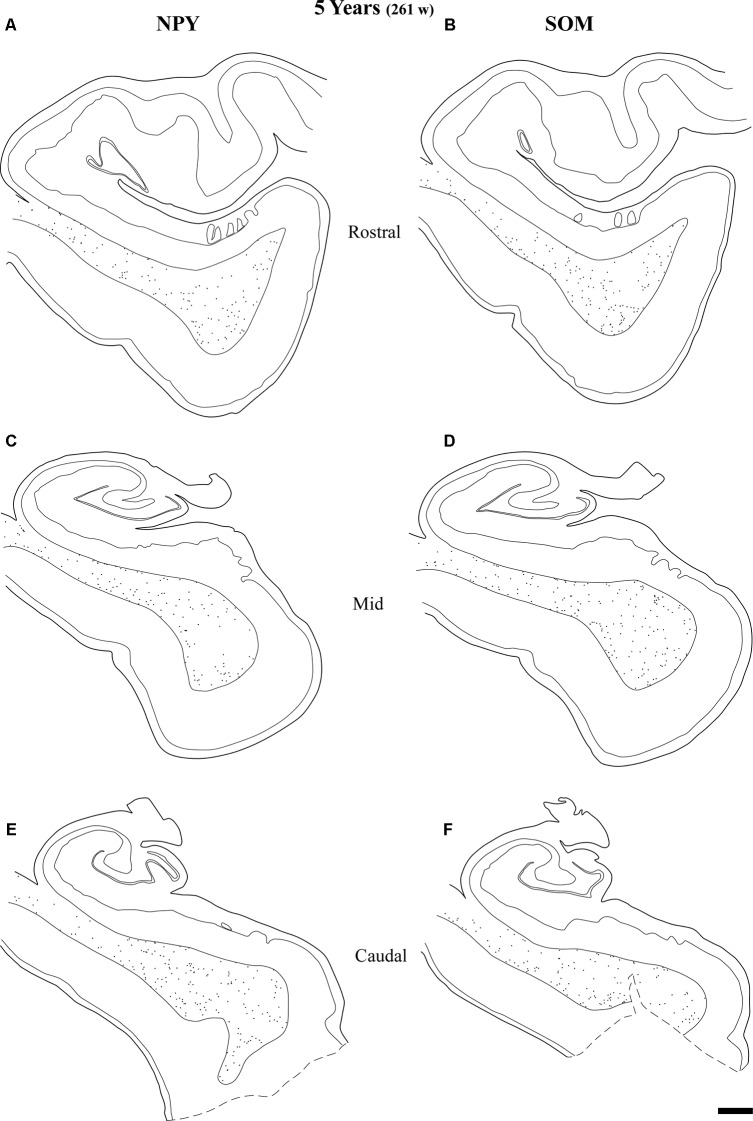
Line drawings with the distribution of NPY- and SOM-28-immunoreactive neurons in the angular bundle of a 5 years old case at rostral **(A,B)**, mid **(C,D)** and caudal **(E,F)** levels. Note the decrease in density at all levels compared to younger ages. Other conventions as in Figure 3. Scale bar is 2 mm.

**Figure 12:**
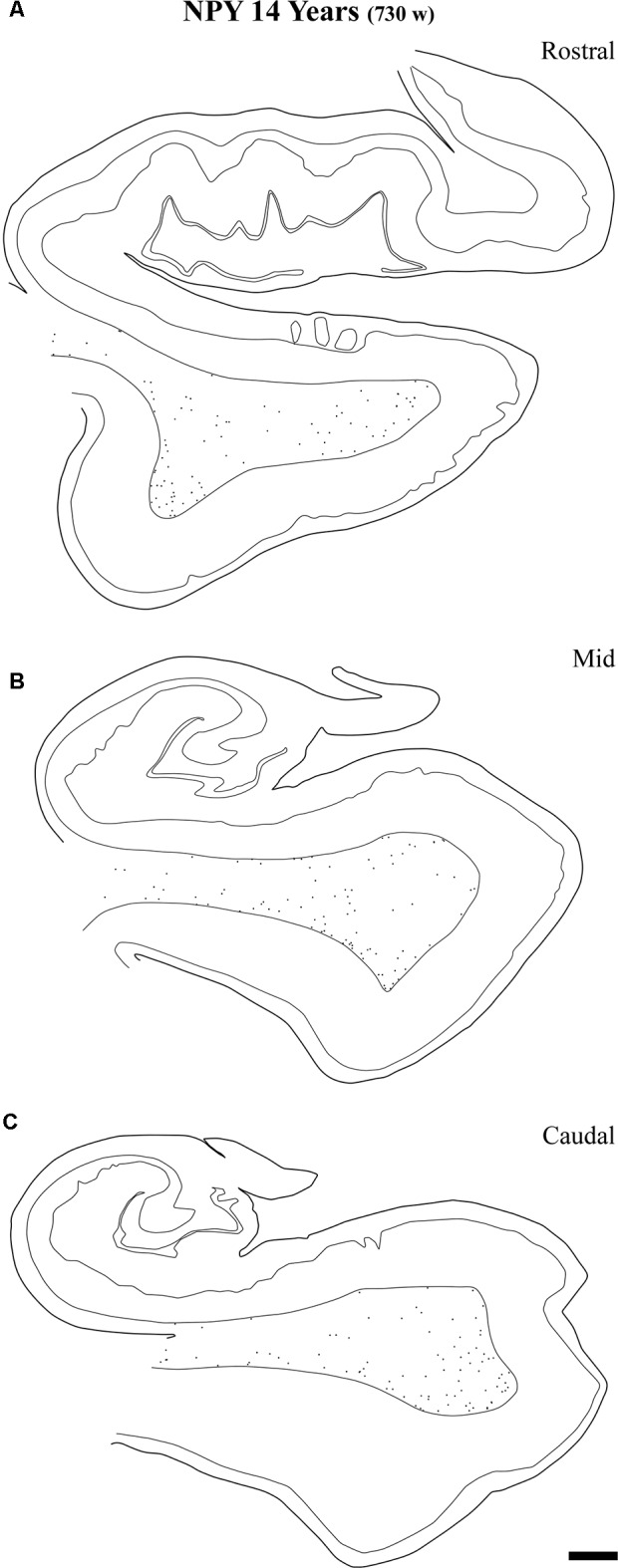
Representation of the NPY-positive labeling in a 14-year-old case at the level of the head **(A)** body **(B)** and tail **(C)** of the angular bundle. The decrease in density at the three topographical levels of the angular bundle is noticeable, in particular compared to ages younger than 23 months. Other conventions as in Figure 3. Scale bar is 2 mm.

**Figure 13:**
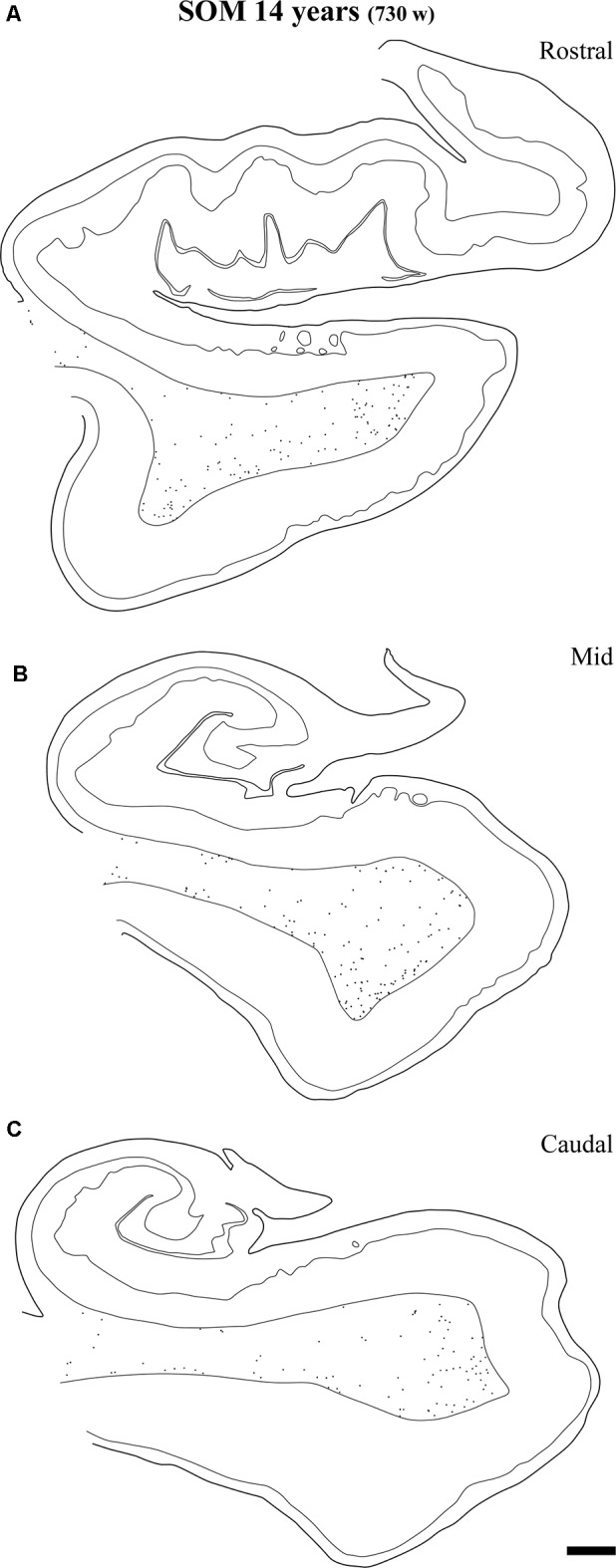
Plots of SOM-28 positive neurons in the angular bundle at rostral **(A)** middle **(B)** and caudal **(C)** levels of the hippocampus at 14 years. Note the decrease in density at the three levels compared to younger ages, at the same time that the remaining neurons localize preferentially near the border of the gray matter with layer VI of the overlying cortex (entorhinal or parahippocampal cortices). Other conventions as in Figure 3. Scale bar is 2 mm.

We observed rostrocaudal differences in the angular bundle situated at the level of the head, body and tail of the hippocampus. The angular bundle boundaries taken as reference are those explained above, which can be summarized as follows: (1) rostral level, by hippocampus and entorhinal-perirhinal cortices; (2) midlevel, by hippocampus and posterior parahippocampal cortex; caudally, hippocampus and posterior part of the parahippocampal cortex. Figure 7 shows the relative density of neurons in the angular bundle which are also represented in the following figures (Figures 8–13) as line drawings at three levels of the rostrocaudal extent of the angular bundle, and across the different ages studied. The number of immunoreactive neurons decreases with age, as it can be noticed by the different scale in density. Namely, the density of neurons containing NPY falls progressively across ages. At birth, the density is about seven neurons per mm^2^, while at 14 years of age the density has dropped to less than two neurons per mm^2^, what results in a decrease of more than 60%. The density of neurons SOM-28-immunoreactive equally presented a decrease at all levels of the angular bundle and ages. At birth, the density almost reaches 15 neurons per mm^2^; at 18 months of age the density had decreased to eight neurons by mm^2^. However, the highest decrease in density was seen already at 5 years of age with a drop to four neurons per mm^2^, with no much variation between 5 years and 14 years (density of less than three neurons per mm^2^). Topographically, the highest decrease in density corresponded to the caudal level, in contrast with NPY cellular density.

Figure 8 represents the results obtained at 40 gw, which show plots of both NPY- and SOM-28-positive neurons in the white matter of the angular bundle. While the density of immunoreactive neurons is high for both peptides at the three levels, it was nonetheless somewhat higher at the most caudal level (Figures 7, 8). Interestingly, a higher density of neurons around the raphe of the temporal horn in the lateral ventricle was observed (Figures 8C–F). This condensation of neurons at this particular location was not observed at the rostral level examined.

Figure 9 shows the same type of plots of NPY-positive neurons at 18 postnatal months (78 postnatal weeks), where the density of neurons has decreased relative to 40 weeks. In particular this decrease was noted at rostral and caudal levels, while at the level of the body of the hippocampus the decrease was less evident (Figure 9, compare level B with levels A and E). At 18 postnatal months (78 postnatal weeks, Figure 10), SOM-28-immunoreactive neurons were present at the three levels studied. A small decrease in the density of positive neurons seemed to be present at the caudal level (Figure 10), while mid and rostral levels presented about the same density, although midlevel showed a slight predominance. The comparison between both peptides at this age (18 postnatal months, 78 postnatal weeks), showed that that at the level of the body of the hippocampus, SOM-28-positive neurons were more numerous in the angular bundle. Figure 11 represents plots of positive neurons for both peptides, NPY and SOM-28 at 5 years of age (261 postnatal weeks). A decrease in density of neurons for both peptides is evident (compare Figures 9, 10 with 11).

At the age of 14 years (730 postnatal weeks), plots depicting the number of cell bodies containing NPY (Figure 12) and SOM-28 (Figure 13) showed a much reduced density in the population of positive neurons for both peptides, although they remained detectable, in particular at the proximity of layer VI of the gray matter. However, the concentration of peptide positive neurons almost disappeared from the raphe of the fused walls of the lateral ventricle; and only remnants of this population persisted at rostral portions of the angular bundle (Figure 11A).

### Distribution of NPY- and SOM-28-Immunoreactive Fibers

Immunoreactivity for both peptides, NPY and SOM-28 formed a meshwork of fibers resembling a backdrop against which immunoreactive neurons were clearly noticeable (Figures 1D, 4C). As stated above, fibers fanned out towards the crown of the gyral surface of the angular bundle, and in this regard, they were parallel in direction to the spindle-shaped neurons referred to above for NPY- and SOM-28-immunoreactive neurons. The density of immunoreactive fibers was higher for SOM-28 than NPY. Both peptides showed a decrease in the density of fibers at increasing ages, in such a way that before the second postnatal year, the reduction was clearly noticeable (compare Figures 1D, 4C,F).

## Discussion

### Methodological Considerations

Some methodological considerations are required for the interpretation of the morphological results obtained in the present work, since different results can be obtained according to the methodology employed as previously described (Herlenius and Lagercrantz, 2001). Fixation protocols are important in the appearance of immunohistochemical identification of peptide positive cells and fibers (Lavenex et al., 2004, 2009). Our previous study on the postnatal development of several neuropeptides in the dentate gyrus (Cebada-Sánchez et al., 2014) indicates that both NPY and SOM-28 are stable because they have a large molecule size and renders a better resistance to post-mortem degradation before fixation. A different issue is the problem of antibody penetration into the tissue. In a previous study of the distribution of NPY-immunoreactive profiles carried out on the hippocampus of children, Lotstra et al. (1989) suggest that the myelination process might have an effect on the degree of NPY-immunoreactivity. However, in our series, we observed maximal immunoreactivity, both in neurons and fibers, up to 23 months, well after myelination occurs in the infant brain (in the angular bundle about 1 year (Graterón Colmenares, 1997), what has also been observed in studies of NPY in the human brain (Uylings and Delalle, 1997). Therefore, our data suggest that the phenomenon of progressive myelination does not hamper the immunoreactivity for both of these peptides.

### Comparison With Previous Studies

A few studies can be found in the literature regarding the immunohistochemical detection of these neuropeptides in the adult human hippocampal formation (Chan-Palay et al., 1986, 1985; Bouras et al., 1987; Amaral et al., 1988). Notably, the work of Kowall and Beal (1988) examines the density of neurons in different cortical areas, including the EC. The distribution and density of SOM-28 and NPY shown in this study are very similar to our oldest age cases. Moreover, these authors make the observation of the resistance to neurofibrillary tangles pathology and their preservation of these interneurons, albeit altered in their morphology. The distributions of NPY and SOM-14 were studied in the infant hippocampal formation by Lotstra et al. (1989). In those reports, both immunoreactive neurons and fibers for the neuropeptides NPY and SOM-14 in the infant hippocampal formation were described, including the adjacent white matter (angular bundle). In the NPY report, their series included nine cases (postnatal ages from 2 days up to 4 years), although information about the postconceptional weeks is not provided; an additional adult case (42 years old) completes the series (Lotstra et al., 1989). In their SOM-14 report, the series comprised postnatal ages from 2 days up to 4 years, without further information on the cases. Both neuropeptides yielded positivity, both in neurons and fibers of the angular bundle, in addition to other hippocampal structures. Our results confirm the presence of neuropeptide-containing neurons, as well as general features of the immunostained neurons. However, those reports do not mention rostrocaudal differences, which we found along the rostrocaudal axis of the angular bundle. Rostrocaudal differences were noticed for both peptides in our material, although they did no match exactly in density along the different ages. Once the adolescence is completed, the number of white matter neurons do not seem to vary significantly (Mortazavi et al., 2016). The significance of this difference is not known, although it could be speculated that from the time of birth up to one and a half years neurons originated in the caudal ventricular zone are progressively incorporated (Seress, 2007). Progressively, they decrease in the angular bundle although in a non-uniform fashion. The decrease in the number of neurons as maturation progresses is a well-known phenomenon (programmed neuronal death), while, in parallel, axonal growth and pruning of dendrites takes place in monkeys (Lavenex et al., 2007). This fact constitutes a phenomenon of protracted development which extends at least until adolescence (Kostović et al., 2014; Dubois et al., 2014).

Lotstra et al. (1989) report the presence of high density of NPY- and SOM-14-immunoreactive fibers in the deep portion of the Subiculum, Presubiculum, Parasubiculum and EC, close to the border with the angular bundle white matter. The occurrence of NPY and SOM-28 in our study confirms the results reported by Lotstra et al. (1989). Our results also confirm the radial disposition of the fibers and the orientation of both NPY- and SOM-28-positive fusiform neurons fanning out the white matter of the angular bundle. Kostović et al. (2014) also report the presence of immunopositive fibers for neuropeptides and other substances organized in radial bundles directed towards the crown of the gyral portions of the cortex (brain circumvolutions or gyri).

A particular comment deserves the presence of NPY and SOM-28 in the angular bundle white matter underneath the line of fusion of the temporal horn of the lateral ventricle. Here, and at all ages but 14 years old, the presence of immunoreactive neurons is noticeable. The report of neurons extending medially under the deep layers of the Subiculum, is mentioned in the analysis of Lotstra et al. (1989). However, in their publication there is no mention of this particular group of neurons, although the presence of neurons in the “deepest layers of the subicular complex and EC” may suggest that they detected neurons near the border with the gray matter.

While it is difficult to advance a significance of this particular disposition of immunopositive neurons and fibers, it could be speculated a relationship between NPY and SOM-28 with the myelination process, a typical postnatal developmental phenomenon. The myelination of the central nervous system can be traced to the end of the third trimester of gestation (Arnold and Trojanowski, 1996). Specifically, the myelination of the angular bundle is present at birth (Graterón Colmenares, 1997), and continues progressively. At 18 months the angular bundle is myelinated (Brody et al., 1987), and other reports mention that the myelination continues until adolescence. At this point, the cellularity decreases and the myelination increases to the point that there is no much difference in the degree of myelination between 16 years and 62 years (Arnold and Trojanowski, 1996). Likewise, (Benes, 1989; Benes et al., 1994) report a dense myelination at 3.5 years (179 postnatal weeks) in the angular bundle white matter, as well as an increase in myelination in the Subiculum and Presubiculum in late adolescence, which remains invariable from 19 years on.

The relationship between NPY and SOM-28 in myelination is little known. Hashimoto et al. (2011) reported NPY effect on myelination. They injected recombinant NPY in mice, which led to the increased of myelinated axons in parietal cortex. (Stanic et al., 2008) reported a decrease in the number of oligodendrocytes and migratory neuroblasts in transgenic mice that lacked NPY receptors. In the same vein, (Laskowski et al., 2007) found fewer oligodendrocytes in one NPY knock-out model of mice. On the other hand, references in the literature on the relationship between SOM and myelination are even scarcer than for NPY. Carpentier et al. (1999) noticed a decrease in myelination associated to a decrease in SOM binding sites.

It can be concluded that, although the role of these two neuropeptides, NPY and SOM-28, are far from being understood, the relationship with the myelination along the postnatal development seems the most plausible one (Suárez-Solá et al., 2009). Kostović et al. (2011) points to the relationship between these two peptides and other postnatal phenomena, such as the balance between excitatory and inhibitory interneurons. The NPY- and SOM-28-positive neurons present in the angular bundle can be considered as interstitial neurons that show the morphology of interneurons and neurotransmitter features of inhibitory GABAergic neurons (Hendry et al., 1984; Jones and Hendry, 1986). Not only inhibitory interstitial interneurons seem to exist in the white matter but excitatory as well, being excitatory the pyramidal-like type of neurons as reported by Suárez-Solá et al. (2009), while the remainder would correspond to inhibitory neurons (Meyer et al., 1992).

Another possible role for these neuropeptides in the angular bundle can be related to the synaptic circuitry they may form (García-Marín et al., 2010). The early origin of those neurons in the subplate, plus their sharp decrease after 2 years of age suggests that these neurons, which also display NPY immunoreactivity (Chun and Shatz, 1989) may have a role in orienting neuroblasts to their final destination.

Finally, an association between NPY and cerebral circulation is considered, as NPY contributes to cerebral innervation, and has been implicated in the autoregulation of cerebral blood flow (Hamel, 2006).

This is the first report on the relative density topographical distribution of neuronal populations containing NPY and SOM-28 in the white matter of the angular bundle in a developmental series of children. The distribution of immunoreactive neurons and fibers show rostrocaudal differences that might be related to the neurogenesis and formation of subplate neurons, whose dispersion along the fiber trajectory of the angular bundle suggests a role in the organization of the whole hippocampal formation, and its main function in autobiographic and spatial memory.

### Functional Considerations

Our results indicate that the population of interstitial neuropeptidergic neurons in the white matter of the angular bundle seems to be transitory, until it reaches a relatively invariant state, approximately from 2 years on. Although myelination is not complete, the function of the medial temporal lobe (after all, the angular bundle is the white matter of the medial temporal lobe), can attain maturity from this age. The timetable of the density of NPY- and SOM-28-immunoreactive neurons is compatible with the timetable of memory development in humans and nonhuman primates (Bachevalier et al., 1993; Bachevalier and Mishkin, 1994; Pascalis and de Schonen, 1994; Pascalis and Bachevalier, 1999). The connectivity between the EC and the dentate gyrus, as well as with other hippocampal fields, is present prenatally, at 20 gw (Hevner and Kinney, 1996), in addition to the maturation of other hippocampal formation components.

The mature function of the HF cannot be expected without a fully developed circuitry of interneuron system with its neuropeptide content. This is crucial for the modulation of neuronal activity and the maintenance of appropriate brain function, which depends on both principal and GABAergic neurons. Neuronal connectivity in the human hippocampus reaches fully developed functionality between the 2nd and 8th years (Bachevalier et al., 1993; Bachevalier and Vargha-Khadem, 2005). The start of the functional maturation of the hippocampal formation starting at 2 years is coincident with our data, which show that the main pattern in the distribution and density of NPY and SOM-28 is established approximately at this age around 2 years. Later on in development, and likely dependent on myelination, the learning of associations between stimuli begins at about 5–6 years, (Bachevalier and Vargha-Khadem, 2005; Abrahám et al., 2007). Our results support that, although different neuropeptides and neurotransmitters are present at birth, the functional maturity of the hippocampal formation and memory function depends on a coordinated and precise timing of postnatal development in humans.

## Author Contributions

SC-S and PR contributed equally to the work. AI contributed in part of the study. RI designed, wrote and prepared the manuscript.

## Conflict of Interest Statement

The authors declare that the research was conducted in the absence of any commercial or financial relationships that could be construed as a potential conflict of interest.

## Footnotes

^1^The term perirhinal cortex is employed generically to mean both, BA area 35 (perirhinal cortex) and BA area 36 (ectorhinal cortex).
